# Toward a chemotherapy and allo-HSCT free future: the evolution of treatment for Philadelphia chromosome-positive acute lymphoblastic leukemia

**DOI:** 10.3389/fimmu.2026.1809757

**Published:** 2026-04-20

**Authors:** Qixing Yang

**Affiliations:** Tongji Medical College, Huazhong University of Science and Technology, Wuhan, China

**Keywords:** acute lymphoblastic leukemia, allogeneic hematopoietic stem cell transplantation, immunotherapy, Philadelphia chromosome, tyrosine kinase inhibitors

## Abstract

The treatment landscape for Philadelphia chromosome-positive acute lymphoblastic leukemia (Ph+ ALL) has undergone a profound transformation over the past two decades. The integration of *BCR-ABL1* tyrosine kinase inhibitors (TKIs) has shifted the paradigm from reliance on intensive chemotherapy and allogeneic hematopoietic stem cell transplantation (allo-HSCT) towards targeted and immunotherapy-based strategies. Imatinib significantly improved initial complete remission (CR) rates and survival, enabling more patients to proceed to transplant. Second-generation and third-generation TKIs further improved outcomes by targeting most imatinib-resistant mutations, with ponatinib-based regimens achieving deep molecular responses and long-term survival in most patients. Concurrently, immunotherapies like blinatumomab and CAR-T cells have enabled potent chemotherapy-free strategies, yielding high molecular response rates and challenging the necessity of allo-HSCT for all patients. Current evidence supports reserving allo-HSCT for high-risk patients, while those with sustained minimal residual disease (MRD) negativity may be cured with TKI and immunotherapy alone. Future progress hinges on optimizing combinations, integrating novel agents like asciminib and venetoclax, and leveraging MRD and genomic profiling for precision medicine.

## 1 Introduction

Philadelphia chromosome-positive acute lymphoblastic leukemia (Ph+ ALL), defined by the t(9;22)(q34;q11) translocation generating the *BCR::ABL1* fusion gene (1), represents a high-risk subtype comprising 20%-30% of adult ALL cases, with incidence rising to 50% in patients aged ≥60 years (2, 3). Historically, the 5-year overall survival (OS) for Ph+ ALL patients was dismal (<20%), even with intensive chemotherapy. While allogeneic hematopoietic stem cell transplantation (allo-HSCT) improved outcomes for some Ph+ ALL patients, its application was limited by transplant-related complications and relapse risk (4). The introduction of imatinib marked the dawn of targeted therapy, with prospective studies demonstrating that its combination with chemotherapy significantly improved complete remission(CR) rates and long-term survival compared to the pre-tyrosine kinase inhibitors(TKI) era and increased allo-HSCT opportunities. Second-generation and third-generation TKIs expanded options against resistant mutations. Concurrently, the rise of immunotherapy enabled “chemotherapy-free” strategies. These advances necessitate a re-evaluation of allo-HSCT. Optimizing transplant timing, indications, and post-transplant strategies are current priorities, guided by refined minimal residual disease (MRD) monitoring, which is pivotal for personalized treatment and improving cure rates. This review will analyze the evolution of Ph+ALL treatment, focusing on TKI progression, immunotherapy breakthroughs, re-evaluation of allo-HSCT, and precision interventions.

## 2 Chemotherapy in combination with TKIs

### 2.1 Intensive chemotherapy plus TKIs

The introduction of TKIs has revolutionized the therapeutic landscape of Ph+ ALL. In the pre-TKI era, chemotherapy alone yielded dismal outcomes, with long-term survival rates in adult patients remaining around 10–20%. Although combining first-generation TKIs like imatinib with chemotherapy significantly improved remission and survival, it also increased induction-phase toxicity and treatment-related mortality. This has driven the generational evolution of TKIs to optimize both efficacy and safety.

#### 2.1.1 Intensive chemotherapy plus imatinib

Clinical studies have established that the addition of imatinib to chemotherapy significantly improves efficacy and long-term outcomes in Ph+ ALL compared to the pre-TKI era (5–7). In a study of 87 newly diagnosed patients receiving continuous imatinib therapy, the CR rate was 94.3%, with 5-year relapse-free survival (RFS) and OS rates of 39.0% and 33.4%, respectively. Among the 56 patients who underwent allo-HSCT in first complete remission (CR1), the 5-year cumulative relapse rate was 59.1%, and OS rate was 52.6% (6).

Prospective trials have further validated the clinical impact of imatinib. The UKALLXII/ECOG2993 trial, the largest prospective study to evaluate imatinib in Ph+ ALL, compared 266 patients treated in the pre-imatinib era with 175 patients who received imatinib-based therapy. The imatinib group demonstrated a significantly higher CR rate (92% vs. 82%, P = 0.004), along with improved 4-year OS (38% vs. 22%, P = 0.003) and event-free survival (EFS) (33% vs. 18%). The risk of relapse was reduced by 41% (odds ratio [OR]=0.59, P = 0.0003). Furthermore, the rate of allo-HSCT increased from 31% to 46% (P = 0.002). Multivariate analysis confirmed that imatinib independently reduced the risk of EFS (hazard ratio [HR]=0.64, P = 0.02). Notably, even patients who did not undergo transplantation showed a trend toward improved 4-year OS (21% vs. 16%, P = 0.06) (4).

Similarly, the Northern Italy Leukemia Group Protocol 09/00 reported higher CR and allo-HSCT rates in the imatinib-treated group (CR: 92% vs. 80.5%; allo-HSCT: 63% vs. 39%), along with significantly improved OS (38% vs. 23%) and EFS (39% vs. 25%) (8).These results demonstrate that imatinib improves initial responses and survival, thereby allowing more patients to undergo allogeneic stem cell transplantation. This represents a primary mechanism for the sustained clinical benefits observed.

#### 2.1.2 Intensive chemotherapy plus dasatinib

The combination of dasatinib with chemotherapy has demonstrated promising efficacy and a favorable safety profile in the treatment of newly diagnosed Ph+ ALL. In a multicenter trial involving 97 patients treated with dasatinib plus chemotherapy, 88% achieved CR or CR with incomplete count recovery (CRi), and 41 patients proceeded to allo-HSCT in CR1. After a median follow-up of 36 months, the 3-year OS, EFS, and RFS rates were 69%, 55%, and 62%, respectively (9).

A key pharmacological advantage of dasatinib is its activity against most imatinib-resistant *BCR::ABL1* mutations (*E255K, Y253H*). Furthermore, its superior penetration of the blood-brain barrier compared to imatinib enables more effective control of central nervous system disease, as demonstrated in preclinical models. This property is clinically relevant, as poor CNS penetration has been associated with isolated CNS relapse in patients receiving imatinib-based therapy (10).

These advantages were further substantiated in a Chinese multicenter phase 3 randomized trial (ChiCTR-IPR-14005706), which compared dasatinib (80 mg/m²/day) with imatinib (300 mg/m²/day) in combination with intensive chemotherapy in 189 pediatric Ph+ ALL patients. With a median follow-up of 26.4 months, the dasatinib group exhibited significantly superior 4-year EFS rate (71.0% vs. 48.9%, P = 0.005) and OS rate (88.4% vs. 69.2%, P = 0.04), along with a significantly lower overall relapse rate (19.8% vs. 34.4%, P = 0.01) and a trend toward reduced isolated CNS relapse (2.7% vs. 8.4%, P = 0.06) (11).

Dasatinib has also shown significant activity in relapsed/refractory (R/R) Ph+ ALL. In a study of 34 patients with R/R Ph^+^ ALL or lymphoid blast crisis chronic myeloid leukemia treated with dasatinib plus Hyper-CVAD, the overall response rate was 91%, with 71% achieving CR (12).

Nevertheless, the long-term efficacy of second-generation TKIs remains limited by the high frequency of T315I mutations-detected in approximately 37% of relapsed cases-and underlying clonal heterogeneity. Dose optimization and toxicity management are therefore essential (13, 14). A phase III trial demonstrated that once-daily dasatinib (140 mg) provided comparable efficacy to twice-daily dosing (70 mg) in terms of major hematologic response (38% vs. 32%), with a lower incidence of pleural effusion (18% vs. 32%). Subsequent studies have confirmed that dose reduction for toxicity management does not compromise treatment response, with advanced age and prolonged treatment duration identified as key risk factors for pleural effusion (15). In pediatric populations, a dasatinib dose of 60 mg/m²/day in combination with chemotherapy has been shown to balance efficacy and safety, achieving a 3-year EFS of 65.5% (16).

#### 2.1.3 Intensive chemotherapy plus ponatinib

The third-generation TKI ponatinib offers advantages over first- and second-generation TKIs. It overcomes resistance mutations, most notably T315I, and establishes a new therapeutic benchmark by enabling deep molecular responses and sustained long-term survival in Ph+ ALL.

In the NCT01424982 trial, 86 patients (median age 46 years) received hyper-CVAD plus ponatinib. Among them, 68 achieved CR, and the cumulative complete molecular response (CMR) rate reached 86%. After a median follow-up of 80 months, the estimated 6-year EFS and OS rates were 65% and 75%, respectively (17). Similarly, in the NCT02776605 trial, all 30 patients treated with ponatinib and chemotherapy achieved hematologic response. With a median follow-up of 2.1 years, 29 patients remained alive, and the 3-year EFS and OS rates were 70% and 96%, respectively (18).

Although no prospective head-to-head trials have directly compared different TKIs combined with chemotherapy in Ph+ ALL, a retrospective analysis of 204 patients treated with hyper-CVAD plus a TKI between 2001 and 2018 demonstrated that the ponatinib group achieved a significantly higher 3-month CMR rate (74%) than the dasatinib (52%) and imatinib (32%) groups (19). Propensity score-matched analysis further revealed superior 3-year EFS (69% vs. 46%, P = 0.04) and OS (83% vs. 56%, P = 0.03) for patients receiving ponatinib compared to those receiving dasatinib (20, 21).

### 2.2 De-intensified chemotherapy plus TKIs

Many Ph+ ALL patients are ineligible for intensive chemotherapy due to advanced age or comorbidities. The efficacy of newer TKIs has prompted reconsideration of whether intensive chemotherapy remains necessary.

#### 2.2.1 De-intensified chemotherapy plus imatinib

The Group for Research on Adult Acute Lymphoblastic Leukemia (GRAALL)compared high-dose imatinib (800 mg/day) with reduced-intensity chemotherapy (arm A) versus standard imatinib plus hyper-CVAD (arm B). Arm A showed a higher CR rate (98% vs. 91%; P = 0.006), while major molecular response rates were comparable (66% vs. 64%). Estimated 5-year EFS and OS rates for arm A were 37.1% and 45.6% (22). A chemotherapy-free regimen of imatinib plus glucocorticoids yielded median survival and median hematologic response duration of 20 and 8 months, respectively, among 29 patients. The 12-month OS and DFS rates were 74% and 48%, respectively (23).

#### 2.2.2 De-intensified chemotherapy plus dasatinib

In the EWALL trial of 71 elderly patients (median age 69), dasatinib plus low-intensity chemotherapy led to a 96% CR rate and 36% 5-year OS rate. 36 patients relapsed, 24 with T315I mutations (24). The Japanese UMIN000012173 study used a two-step induction: dasatinib plus prednisolone resulted in CR/CRi in all 78 patients, followed by dasatinib plus intensive chemotherapy, achieving MRD negativity in 52.6%. HSCT was performed in 74.4% of patients, with 3-year EFS and OS of 66.2% and 80.5% (25).

A multicenter prospective trial in adults with newly diagnosed Ph+ ALL evaluated dasatinib plus dexamethasone induction, achieving a 98.5% CR rate with no early mortality. After a median 59-month follow-up, 5-year DFS and OS rates were 37% and 48%, respectively. Patients who underwent reduced-intensity conditioning allo-HSCT had superior outcomes (5-year OS: 62%). The p210 *BCR-ABL1* isoform was associated with significantly poorer prognosis compared to p190 *BCR-ABL1*. The T315I mutation was the primary driver of bone marrow relapse, while dasatinib’s limited CNS penetration may contribute to CNS recurrence. Pharmacokinetic analysis revealed a median CSF/plasma ratio of 0.66% (median plasma concentration: 52.4 ng/mL; CSF: 0.348 ng/mL). Calculations using the dissociation constant for drug binding to α1-acid glycoprotein suggest that 70%–97% of dasatinib in the CSF exists in the free form, whereas only 1%–3% is unbound in plasma. This indicates that CSF levels are governed by free plasma drug. Analysis of 39 patients without active CNS disease confirmed that dasatinib’s CSF penetration is comparable to that of imatinib, suggesting this pharmacological limitation underpins CNS relapse in a subset of patients (26).

Dasatinib-based chemotherapy-free regimens showed high CR rates (97–100%), but relapse rates reached 74% when TKIs alone were continued post-remission (27, 28).

#### 2.2.3 De-intensified chemotherapy plus ponatinib

The INCB84344–201 trial evaluated ponatinib plus prednisone and intrathecal chemotherapy. Among 44 patients, 95.5% achieved complete hematologic response (CHR), 81.8% CMR, and median EFS was 14.31 months (29). Sequential ponatinib with mini-hyper-CVD and blinatumomab resulted in CR in all 20 patients; 2-year continuous remission duration (CRD) and OS rates were 93% and 83%, respectively (30, 31). A phase III trial in 245 patients (median age 54) showed a significantly higher MRD-negative CR rate with ponatinib versus imatinib combined with low-intensity chemotherapy (34.4% vs. 16.7%; P = 0.002), with comparable safety (32). Quality-Adjusted Time Without Symptoms of Disease or Toxicity (Q-TWiST) analysis indicated ponatinib improved quality-adjusted survival over imatinib (relative gain 10.98%; p = 0.015) (33).

Despite its efficacy, ponatinib requires careful safety management. Common grade 3–5 adverse events included infection (93%), elevated liver enzymes (31%), and hypertension (17%). Arterial occlusive events represent a major concern, though their incidence was overestimated due to non-standardized reporting. Adjudicated analysis of the PACE trial revealed an arterial occlusive event rate of 17%, significantly lower than the unadjudicated 25%, with exposure-adjusted rates declining over time. Among patients experiencing arterial occlusive events, 81% continued therapy; only 9% discontinued due to the event. Patients with ≥3 baseline cardiovascular risk factors had significantly higher arterial occlusive event risk (29%) compared to those with none (3%). A dose-titration strategy (starting at 45 mg and reducing to 15 mg based on response) has been shown to mitigate cardiovascular risk while maintaining efficacy. Clinical application should emphasize cardiovascular risk assessment and individualized dose optimization (34, 35).

#### 2.2.4 De-intensified chemotherapy plus novel TKI-asciminib

Asciminib, an allosteric ABL1 inhibitor, targets the myristoyl pocket of BCR-ABL1. Unlike conventional TKIs, it preserves CD20 expression and NK/macrophage effector functions, making it suitable for combination with anti-CD20 antibodies (36). In a phase I trial, asciminib plus dasatinib and prednisone led to an 84% 28-day hematologic CR rate and 89% 84-day MRD negativity rate, with good tolerability in older patients (37). Data in Chinese patients showed asciminib (median 240 mg/day) induced CMR or deep response in 60% of resistant cases, with a favorable safety profile (38).A retrospective study of 41 patients with R/R Ph+ ALL/LBC-CML treated with Asciminib (mono- or combination therapy) showed a CR/CRi rate of 83% and a CMR rate of 56.5%. Median OS was 9.8 months; combination therapy yielded longer EFS than monotherapy (7.93 vs. 4.23 months). Treatment was well-tolerated with no discontinuations (39).

**Table 1** summarizes prospective trials in adult Ph+ ALL in the TKI era by chemotherapy intensity.

**Table 1:** Frontline trials of TKI-based regimens in Ph+ ALL patients.

TKI	N	Patient age, median (range), y	Overall CMR rate, %	CHR rate,%	Allogeneic SCT rate, %	RFS rate,% (y)	OS rate,% (y)	Reference
Intensive chemotherapy + TKI								
Imatinib	175	42 (16-64)	NA	92	46	50 (4)	38 (4)	Fielding, 2014
Imatinib	87	41 (16-71)	NA	94	64	39 (5)	33 (5)	Lim,2015
Imatinib	54	51 (17-84)	45	93	30	43 (5)	43 (5)	Daver,2015
Dasatinib	94	44 (20-60)	20	94	44	62 (3)	69 (3)	Ravandi, 2016
Dasatinib	92	7.8 (5.2-11.3)	NA	98	NA	71 (4)	88 (4)	Shen,2020
Dasatinib	65	60 (22-87)	98.5	NA	28	37 (5)	48 (5)	Matthew,2021
Ponatinib	86	46 (21-80)	86	100	23	65 (6)	75 (6)	Kantarjian,2023
Ponatinib	30	49 (19-59)	71	100	87	70 (3)	96 (3)	Ribera, 2022
Olverembatinib	40	39 (30-48)	55	92	32	80 (1)	93 (1)	Wen,2025
Low intensity chemotherapy + TKI								
Imatinib	133	47 (18-59)	29	98	62	37 (5)	46 (5)	Chalandon, 2015
Dasatinib	78	44.5 (16-64)	NA	100	74	66 (3)	81 (3)	Sugiura,2022
Dasatinib	13	41 (16-73)	15	100	46	46 (2)	91 (2)	Li, 2020
Dasatinib	60	41.9 (18.7-59.1)	18	97	37	51 (1.7)	69 (1.7)	Chiaretti, 2021
Ponatinib	44	66.5 (26-85)	41	95.5	14	32 (2)	66 (2)	Martinelli,2022
Ponatinib	154	54 (19-82)	34	94	30	70 (2)	80 (2)	Jabbour,2024
Olverembatinib	79	42 (15-74)	62	98	30	89 (1)	93 (1)	Gong,2025

CMR, complete molecular remission; CHR,complete hematologic response;NA, not applicable; OS, overall survival; RFS, relapse-free survival; SCT, stem cell transplantation.

## 3 Immunotherapy and targeted agents

Although chemotherapy combined with TKIs has improved the prognosis of Ph+ ALL, persistent challenges—including drug resistance, toxicity, and reliance on transplantation—remain unresolved. In response, immunotherapy is rapidly emerging and, together with novel targeted agents, is profoundly shifting the treatment paradigm toward “chemotherapy-free” and “transplant-free” approaches.

### 3.1 Bispecific T-cell engager: blinatumomab

Blinatumomab is a bispecific T-cell engager that targets both CD3 and CD19. Chemotherapy-free strategies combining TKIs with blinatumomab have shown considerable promise.

In a phase II trial (NCT02143414) involving older patients (≥65 years; P < 0.05), a chemotherapy-free strategy consisting of dasatinib plus prednisone induction followed by blinatumomab resulted in a 3-year OS of 87% and disease-free survival (DFS) of 77% after a median follow-up of 2.7 years. Only one patient underwent allo-HSCT (40).

The GIMEMA LAL2116 (D-ALBA) trial investigated a chemotherapy-free regimen in Ph+ ALL adult patients, utilizing dasatinib as induction therapy followed by blinatumomab as consolidation. Among 63 newly diagnosed patients (median age 54 years), the CR rate reached 98%. The molecular response rate was 29% after dasatinib induction (day 85), which increased to 60% following two cycles of blinatumomab, and further rose to 81% after subsequent cycles. At a median follow-up of 18 months, OS rate was 95%, and DFS rate was 88%. At a median follow-up of 53 months, DFS, OS, and EFS rates were 75.8%, 80.7%, and 74.6%, respectively. Among 29 patients who achieved molecular response after treatment (93.1%), only TKI maintenance therapy was administered without further chemotherapy or transplantation, and 28 of these patients maintained long-term continuous complete hematologic remission. Allo-HSCT in CR1 was primarily performed in patients with persistent MRD, with a continuous complete hematologic remission rate of 83.3% and transplant-related mortality rates of 12.5% (in first remission) and 13.7% (overall). A total of 9 relapses and 6 deaths occurred, with ABL1 mutations detected in 7 cases (41, 42).

Further phase II trials indicate that the combination of ponatinib and blinatumomab in newly diagnosed Ph+ALL yields a CMR rate of 87% and a 3-year OS rate of 91%, with only 3% of patients requiring allo-HSCT. This represents a shift away from the conventional treatment paradigm based on chemotherapy and transplantation (43).

Additionally, a prospective study evaluated the third-generation TKI olverembatinib combined with blinatumomab in 13 patients. All patients achieved CR after one cycle and CMR within three months. The 6-month OS and event-free survival (EFS) rates were 100% and 87.5%, respectively, with one death attributable to relapse after transplantation (44).

In the R/R setting, the ALCANTARA trial demonstrated that single-agent blinatumomab induced a CR rate of 36%, with 88% of responders achieving MRD clearance. Median OS was extended to 19.8 months, corresponding to a 23% reduction in the risk of death compared with conventional chemotherapy (hazard ratio [HR] = 0.77) (45, 46).For R/R Ph+ ALL patients, the combination of blinatumomab and ponatinib achieved a CMR rate of 79% and a 1-year OS of 79%. In terms of safety, grade 3–4 adverse events were primarily infections (37%) and elevated amylase/lipase levels (8%). Treatment discontinuation due to arterial occlusive events (5%) or neurotoxicity (2%) was uncommon. Overall, the regimen was better tolerated than traditional chemotherapy (47).

### 3.2 Chimeric antigen receptor T-cell therapy

Chimeric Antigen Receptor T-cell (CAR-T) therapy is an engineered form of adoptive immune cell treatment based on genetic recombination technology. It typically consists of an extracellular antigen-binding domain linked to one or more intracellular T-cell signaling domains. *In vitro* studies have demonstrated that CD19-specific T-cell exhibit potent activity against TKI-resistant Ph+ ALL cells, including those harboring the T315I mutation (48).

In a single-center phase I trial (NCT03984968), multi-cycle sequential administration of anti-CD19 CAR-T cells, together with autologous CD19^+^ feeder T cells (FTCs) and a TKI, was evaluated as consolidation therapy. At a median follow-up of 27 months, the CMR rate among 15 Ph+ CD19+ B-cell ALL patients was 92%, with RFS and OS rates of 84% and 83%, respectively. CAR-T cells persisted for up to 40 months and effectively eradicated the T315I mutation, suggesting the potential of this combination strategy to replace allo-HSCT in selected cases (49).

A multicenter retrospective trial conducted in China enrolled 93 patients with relapsed/refractory Ph+ B-cell ALL and evaluated CD19-directed chimeric antigen receptor T-cell therapy. The results demonstrated an overall CR rate of 87.1%, with 96.3% of responders achieving flow cytometry-negative MRD status and a complete molecular response rate of 63.4%. After a median follow-up of 25.4 months, the median OS and DFS were 20.8 months and 8.1 months, respectively. Notably, allo-HSCT following CAR-T therapy did not independently improve OS or DFS. The incidence of grade ≥3 cytokine release syndrome and neurotoxicity were 14.0% and 2.2%, respectively, with manageable hematologic toxicity (50).

A sequential regimen combining dasatinib with CD19 and CD22 CAR-T cell infusion demonstrated that among 28 patients with newly diagnosed Ph-positive ALL, the CMR rate was 25% after induction therapy, increased to 85% following CD19 CAR-T therapy, and was maintained at 76% after subsequent CD22 CAR-T treatment. With a median follow-up of 23.9 months, the 2-year overall survival and leukemia-free survival rates were both 92%, and treatment-related toxicities were mild (51).

Another single-center phase 1b/2 trial(NCT02772198) investigated point-of-care anti-CD19 CAR-T therapy in adults with relapsed/refractory B-cell ALL (including 6 Ph+ ALL patients). The overall response rate was 88%, with 81% achieving CR, and 63% attaining MRD-negative CR. The 2-year OS and progression-free survival (PFS) rates were 62% and 56%, respectively (52).

Several trials have explored CAR-T therapy as a bridge to allo-HSCT. For instance, 19-28z CAR-T treatment induced an 88% CR rate in 16 patients with R/R B-ALL; 44% (7 patients) subsequently underwent successful allo-HSCT and remained relapse-free during a median follow-up of 2–24 months (53).A retrospective analysis of 56 patients reported a CR rate of 91.1% following CD19 CAR-T therapy, with 67.9% achieving MRD negativity. Transplanted patients (n=30) exhibited significantly superior 2-year OS (58.9% vs. 22.7%, p=0.005) and leukemia-free survival (53.2% vs. 18.8%, p=0.000) compared to the non-transplanted group (n=21) (54).

Similarly, a prospective single-center trial (NCT03919240) comparing CD19 CAR-T bridging to allo-HSCT (n=17) versus conventional salvage therapy (n=23) in R/R Ph+ALL demonstrated improved 2-year OS (58.8%) and event-free survival (EFS, 52.9%) in the CAR-T cohort (55).

### 3.3 Antibody-drug conjugate: inotuzumab ozogamicin

Inotuzumab ozogamicin (InO) is an intravenous antibody-drug conjugate composed of an anti-CD22 monoclonal antibody linked to the cytotoxic agent calicheamicin. Upon binding to CD22 on the cell surface, the complex is internalized, leading to intracellular release of calicheamicin, which induces double-strand DNA breaks and subsequent apoptosis (56).

Several clinical studies have evaluated the efficacy of InO in patients with relapsed/refractory Ph+ ALL. In a phase 1/2 trial investigating InO combined with the tyrosine kinase inhibitor bosutinib (maximum tolerated dose: 400 mg/day), 83% (15/18) of patients achieved CR/CRi. Among these responders, 61% (11/18) attained flow cytometry-based minimal residual disease (MRD) negativity, and 56% (10/18) achieved CMR. The median overall survival was 13.5 months (57).

Furthermore, a randomized controlled trial demonstrated a superior CR/CRi rate in the InO treatment group (73%, 16/22) compared to the standard chemotherapy group (56%, 15/27) (58).

Another trial evaluated a regimen combining olverembatinib with InO as a bridging therapy to allo-HSCT. The results demonstrated that all 14 patients achieved CR, with a complete molecular response rate of 78.6%. The 2-year OS and RFS rates were 83.3% and 62.9%, respectively. Notably, 64.3% of patients successfully bridged to transplantation, with a 100-day post-transplant mortality rate of 0%. These findings suggest that this chemotherapy-free combination regimen can achieve deep molecular responses and improve transplant outcomes in high-risk patients. Despite the limited sample size, it offers a highly promising new strategy for the treatment of relapsed/refractory or MRD-positive Ph+ALL (59).

### 3.4 BCL-2 inhibitor: venetoclax

Preclinical trials demonstrate strong synergy between BCL-2 inhibitors and TKIs or MCL-1 inhibitors in high-risk B-ALL. In Ph+ ALL, TKIs enhance venetoclax via LYN inhibition, BIM upregulation, and MCL-1 suppression. Dual BCL-2/MCL-1 targeting shows superior efficacy, eradicating leukemia in models. This combination disrupts critical BCR-ABL1/STAT5 survival signals, effectively eliminating leukemic cells (60, 61).

In a phase 1/2 trial evaluating ponatinib, venetoclax, and dexamethasone in nine high-risk patients, the subgroup receiving venetoclax (800 mg/day) plus ponatinib (45 mg/day) (n=6) achieved a composite CR/CRi rate of 83%, and 44% (4/9) attained CMR. All responders maintained remission without proceeding to allo-HSCT. The 1-year overall survival rate was 72% at a median follow-up of 13.2 months (62).The regimen of olverembatinib combined with venetoclax and reduced-intensity chemotherapy demonstrated significant efficacy in newly diagnosed patients (n=79), with a 3-month CMR rate reaching 62%. At a median follow-up of 12 months, the 1−year OS rate was 93.1% and the 1−year EFS rate was 89.1% (63).

A retrospective analysis of the VPD regimen-consisting of venetoclax (100 mg day 1, 200 mg day 2, 400 mg days 3-28), ponatinib (45 mg days 1-28), and dexamethasone (0.15 mg/kg days 1-21, 0.075 mg/kg days 22-28)-reported a CR/CRi rate of 89.5% and a major molecular response (MMR) rate of 57.9% in patients with T315I or compound mutations (64).

Additionally, a phase I trial investigating venetoclax combined with InO in relapsed/refractory ALL showed that all nine enrolled patients achieved CR, with 87% attaining MRD negativity (65).

The following is a summary of the latest immune-targeted treatment strategies (**Table 2**).

**Table 2:** Summary of Immune-targeted therapy for Ph + ALL patients.

Interventions	N	Patient age, median (range), y	Overall CMR rate, %	CR rate,%	Allogeneic SCT rate, %	RFS rate,% (y)	OS rate,% (y)	Reference
blinatumomab	24	73 (65-87)	89	92	NA	77 (3)	87 (3)	Advani,2023
blinatumomab	60	55 (20-83)	83	97	3	77 (3)	91 (3)	Kantarjian,2024
blinatumomab	13	NA	85	100	NA	87 (0.5)	100 (0.5)	Zhang,2023
blinatumomab	63	54 (24-82)	98	NA	48	75.8 (4.4)	80.7 (4.4)	Foà,2024
CAR-T	13	41 (27-58)	92	100	NA	84 (2)	83 (2)	Chen, 2023
CAR-T	27	33 (19-77)	NA	81	NA	56 (2)	62 (2)	Fried,2023
CAR-T	56	34 (18-59)	NA	91	53	53 (2)	59 (2)	Gu,2021
CAR-T	28	48 (18-69)	85	NA	NA	92 (2)	92 (2)	Zhang,2025
CAR-T	93	41 (6-76)	78	87	21	42 (1)	66 (1)	Yu,2026
inotuzumab ozogamicin	26	46 (19-70)	NA	89	23	54 (2)	60 (2)	Jabbour,2024
inotuzumab ozogamicin	18	62 (19-74)	56	83	33	30 (1)	50 (1)	Jain, 2021
venetoclax	9	37 (26-73)	44	56	67	100 (0.5)	72 (1)	Short,2021

CMR, complete molecular remission; CR:complete remission; NA, not applicable; OS, overall survival; RFS, relapse-free survival; SCT, stem cell transplantation.

Despite the remarkable efficacy demonstrated by these immunotherapies and targeted agents in clinical trials, their global implementation is substantially hindered by disparities in accessibility. Significant differences persist in the availability of novel TKIs, blinatumomab, CAR-T cells, and inotuzumab ozogamicin across regions. Constrained by delays in regulatory approval, limited health insurance coverage, and high production costs, many patients in low- and middle-income countries have yet to benefit equitably from these breakthroughs. Nevertheless, as patents expire, generic formulations become available, manufacturing processes improve, and clinical evidence accumulates, the reach of these therapies is expected to expand progressively. Through global collaboration, policy initiatives, and the dissemination of specialized expertise, advanced treatment strategies currently confined to a limited number of centers may be translated into standard care across broader regions. Such efforts are essential to narrowing the survival gap and advancing Ph+ ALL treatment from scientific breakthrough toward global health equity.

## 4 The evolving role of allo-HSCT

The role of allo-HSCT in the management of Ph+ ALL has evolved considerably with therapeutic advances. Early trials established that allo-HSCT significantly improved RFS compared with chemotherapy alone in the pre-imatinib era. Following the introduction of TKIs, a multicenter U.S. trial confirmed that chemotherapy combined with dasatinib followed by allo-HSCT was both well tolerated and highly effective, of the 41 patients who underwent allo-HSCT in first complete remission (CR1), the 12-month EFS and OS post-transplant were 71% and 87%, respectively (9).

In multivariate analysis, allo-HSCT was associated with improved OS (aHR: 0.54; 95% CI: 0.30–0.97; p = 0.04) and RFS (aHR: 0.21; 95% CI: 0.12–0.38; p < 0.001), with 5-year adjusted OS and RFS rates of 73% and 70% in the transplant cohort versus 50% and 20% in the non-transplant cohort (66).

The NCT02776605 trial further demonstrated that ponatinib combined with standard chemotherapy, followed by allo-HSCT in adults under 60 years with newly diagnosed Ph+ ALL, resulted in a 100% hematologic complete remission rate and a 71% rate of CMR by the end of consolidation. A high proportion of patients (87%, 26/30) proceeded to transplantation, with 3-year EFS and OS rates of 70% and 96%, respectively (18).

However, the introduction of chemotherapy-free and immunotherapy-based regimens has prompted reevaluation of the universal need for transplantation. A study of dasatinib, prednisone, and blinatumomab in 24 older patients reported that only one patient required allo-HSCT (40).Similarly, in the NCT01424982 trial, the combination of ponatinib and hyper-CVAD yielded high molecular response rates and survival outcomes in the majority of Ph+ ALL patients without mandating transplantation. Transplantation in CR1 did not significantly affect outcomes (17). The NCT03263572 trial also reported an 87% CMR rate with ponatinib and blinatumomab, without the need for allo-HSCT in first remission (47).

A meta-analysis of 39 studies involving 5, 054 adult patients with Ph+ ALL suggested that among those achieving CMR, survival was comparable between the non-transplant and transplant subgroups. This finding indicates that MRD-guided, TKI-based therapy may allow selected patients to avoid transplantation (67).A large multicenter retrospective study (n=230) further found that although allo-HSCT significantly reduced the cumulative incidence of relapse (CIR: aHR = 0.32, p < 0.001), this advantage was counterbalanced by increased non-relapse mortality (NRM: aHR=2.59, p=0.003), resulting in no significant difference in OS (5-year: 67% vs. 59%; aHR=1.05, p=0.86) or RFS (5-year: 63% vs. 52%; aHR=0.86, p=0.53) between groups. The authors concluded that allo-HSCT in CR1 did not confer a survival benefit for patients who achieved CMR (BCR::ABL1 < 0.01%) within 90 days after induction therapy (68).

Based on current evidence, we propose that indications for allo-HSCT should be individualized and risk-adapted. Transplantation remains the preferred option for patients who fail to achieve CMR within three months of induction, those with high tumor burden, T315I mutations, or persistent MRD after chemotherapy and TKI. By contrast, patients who attain early CMR and tolerate TKIs-particularly when combined with blinatumomab or other immunotherapies-may avoid transplantation-related risks through intensive TKI maintenance. Future approaches should integrate dynamic MRD monitoring, genomic risk profiling, novel therapies, and prospective randomized trials comparing transplantation with TKI plus immunotherapy to better identify patients most likely to benefit from each strategy.

Another area of ongoing investigation is the use of TKIs following allo-HSCT. A national retrospective cohort study (n=850) found that while TKI prophylaxis did not reduce relapse or improve OS in the overall cohort, subgroup analysis suggested a potential benefit with dasatinib (HR = 0.34, p=0.140) but not with imatinib (69). However, post-transplant TKI therapy has been associated with significant toxicity, often necessitating dose modifications and discontinuation (70). Nonetheless, several studies indicate that early initiation of prophylactic TKI (for ≥2 years) after allo-HSCT, guided by dynamic MRD and BCR::ABL1 mutation analysis, can improve survival (5-year OS: 46-84%) and reduce relapse. In a retrospective study of 42 patients in CMR before and 3 months after transplantation, those starting TKI within 3 months post-HSCT (n=18) had significantly superior 2-year progression-free survival (94.5% vs. 75%, p=0.041), supporting maintenance for at least two years (71).

A prospective multicenter study (n=110) compared MRD-positive patients receiving post-HSCT TKI prophylaxis (n=38) with MRD-negative controls not given prophylaxis (n=72). The 4-year cumulative incidence of relapse, non-relapse mortality, and leukemia-free survival were comparable between groups, suggesting that prophylactic TKI can yield outcomes in pre-HSCT MRD-positive patients similar to those in pre-HSCT MRD-negative patients (72).Another analysis of 92 patients receiving TKI for >3 months post-transplant found no difference in RFS between planned and MRD-triggered initiation. Among patients discontinuing TKI before relapse, treatment duration exceeding six months trended toward better RFS (HR = 0.30, p=0.08). A composite endpoint of “current TKI discontinuation without relapse” (cTRFree) was proposed, with a 35% achievement rate at 5 years, supporting the feasibility of TKI cessation in patients without sustained MRD (73).

## 5 Molecular mechanisms and advances in precision therapy

With the in-depth characterization of the genomic landscape of Ph+ ALL, key genomic aberrations such as *IKZF1* and *CDKN2A/2B* deletions, along with their associated signaling pathways and metabolic reprogramming mechanisms, have been progressively elucidated. These findings not only provide a molecular basis for prognostic stratification and treatment selection but also establish a foundation for developing novel targeted strategies against driver mutations and dysregulated pathways.

### 5.1 Key genomic aberrations

Genomic aberrations in Ph+ ALL provide critical insights for prognostic stratification and treatment selection. *IKZF1* deletions are present in approximately 78.8% of patients and are associated with reduced rates of early molecular response and increased relapse risk, although DFS may ultimately be comparable to that of *IKZF1*-wildtype patients (74).In contrast, *CDKN2A/2B* deletions represent a well-established independent poor prognostic factor. Affected patients frequently present with high white blood cell counts, hepatosplenomegaly, and upregulated CD20 expression, leading to lower rates of CMR, increased cumulative incidence of relapse, and significantly shorter OS and DFS (75, 76).

Studies have identified significant upregulation of the oncogenic transcription factors ERG and c-MYC in *BCR::ABL1*-driven B-ALL (77). Leukemic cell growth in Ph+ ALL is highly dependent on MYB-mediated transcriptional activation of CDK6, cyclin D3, and BCL-2 (78). Additional molecular features include overexpression of the glucose transporter SLC2A5 in pediatric Ph+ ALL and upregulation of lysosomal-associated protein LAPTM4B, which is also observed in other malignancies (79, 80).Immunologically, Th17 cells and IL-17A expression are elevated in Ph+ B-ALL; IL-17A activates multiple signaling pathways including *BCR-ABL*, IL6/JAK/STAT3, and NF-κB (81). The *IL7R-CXCR4* molecular platform promotes disease progression, and anti-*IL7R* antibodies have demonstrated efficacy in eradicating Ph+ ALL cells, including resistant populations (82). SHP2 has been identified as essential for *BCR::ABL1*-induced leukemogenesis, suppressing *MXD3/4* through SFK and ERK pathways to promote *Myc*-dependent proliferation (83).

*BCR::ABL1* isoforms drive distinct disease phenotypes: p210 primarily induces chronic myeloid leukemia (CML)-like disease, whereas p190 causes more aggressive Ph+ ALL. This divergence is linked to isoform-specific signaling and metabolic reprogramming. STAT6 is specifically activated in Ph+ ALL through *JAK2*-mediated phosphorylation, promoting leukemogenesis via *c-Myc* transcriptional upregulation. STAT6 inhibition significantly suppresses tumor growth in Ph+ ALL models but has limited effect in CML (84). STAT5, a key downstream effector of *BCR::ABL1* and JAK2 signaling, inhibits apoptosis by regulating *PIM-1*, *MCL1*, and *BCL-2* expression. STAT5 silencing upregulates the pro-apoptotic protein BIM and can reverse TKI resistance, highlighting potential therapeutic targets for overcoming treatment failure (85).

### 5.2 Emerging targeted strategies: signaling pathways

In cell cycle regulation, the *CDK4/6* inhibitor palbociclib (PD0332991) exhibits specific anti-leukemic activity by inducing Rb dephosphorylation and downregulating S-phase gene expression, leading to G0-G1 cell cycle arrest. This effect is mediated through transcriptional and post-transcriptional modulation of *CDK4* and cyclin D2, likely under *BCR::ABL* control via the PI3K pathway (86).

Evidence indicates that JNK pathway activation persists in Ph+ B-ALL and is resistant to *BCR::ABL* TKI monotherapy. The synergistic effect of the JNK inhibitor JNK-IN-8 and dasatinib, demonstrated by significantly improved survival in preclinical models, validates the strategy of cross-pathway combination therapy (87).Apoptosis pathway targeting has also shown promise: although BCL-2 expression is downregulated in Ph+ ALL, compensatory upregulation of BCL-W and BCL-XL occurs. This has prompted evaluation of navitoclax (a BCL-2 inhibitor) combined with MDM2 inhibitors for enhanced synergy (88). Furthermore, dual inhibition of BCL-2 and MCL-1 effectively counteracts these compensatory mechanisms and demonstrates significant efficacy in high-risk B-ALL subtypes, including Ph+ and Ph-like ALL (61).

Additionally, selective *HDAC1/2* inhibitors disrupt *Mre11*-mediated DNA double-strand break repair through chromatin remodeling, synergizing with doxorubicin to block *BCR::ABL1*-driven homologous recombination repair dependency-offering novel strategies to overcome treatment resistance (89). Research into resistance mechanisms further indicates that AKT/mTOR pathway activation is a central driver of non-mutational *BCR::ABL1* resistance; the mTORC1/2 inhibitor Torin-1 and dual PI3K/mTOR inhibitor NVP-BEZ235 effectively reverse this phenotype (90).The immunomodulatory agent lenalidomide specifically degrades the *IK6* isoform, restoring *IKZF1* function, and synergizes with TKIs to induce apoptosis in *IK6*-positive Ph+ ALL, providing a precision therapy option for this molecular subtype (91).

## 6 Monitoring of MRD

Minimal residual disease (MRD) assessment provides critical prognostic information in Ph+ ALL, guiding treatment decisions and transplantation strategies.

Current clinical MRD monitoring primarily employs multiparametric flow cytometry (MFC), quantitative reverse transcription polymerase chain reaction (RT-qPCR), and high-throughput next-generation sequencing (NGS).

A cohort study of 76 patients with Ph+ ALL treated with chemotherapy and a TKI found that achieving both MMR and MFC-negative status at 3 months was associated with reduced relapse and prolonged OS. Notably, achieving immunoglobulin heavy chain (IGH) negativity by PCR did not correlate with improved outcomes, possibly due to the use of consensus rather than patient-specific primers (92).Another study demonstrated a strong correlation between BCR::ABL1 transcript levels (PCR-MRD) and a standardized flow cytometry method (FCM-MRD) (r = 0.7801, p < 0.001), with 88% agreement (κ = 0.761) (93).

The GRAAPH-2014 trial, monitoring 264 Ph+ ALL patients, revealed a key limitation of BCR::ABL1-based MRD: 43% of patients had BCR::ABL1 transcripts in non-leukemic cells, a finding not associated with worse DFS or OS. In contrast, MRD positivity (≥0.01%) measured by immunoglobulin/T-cell receptor (IG/TR) gene markers after treatment cycles 2 and 4 significantly predicted inferior DFS. Multivariate analysis defined a high-risk group by IG/TR positivity after cycle 2 plus high baseline WBC, showing a 4-year DFS of 56.5% vs. 87.6%; allo-HSCT significantly improved outcomes in this group (94). Supporting this, a pediatric study found only 69% concordance between methods, with BCR::ABL1 yielding more false positives, confirming IG/TR’s superior prognostic reliability (95).

A comparative trial between RT-PCR and a high-sensitivity NGS assay (10^-6^) revealed discordant MRD results in 32% of Ph+ ALL patients. Notably, patients who were NGS-negative but PCR-positive exhibited a very low relapse risk, with outcomes comparable to double-negative patients. In contrast, NGS-positive status warranted more intensive treatment. This superior risk stratification by NGS may be explained by the “CML-like biology” in some cases, where BCR::ABL1 transcripts from non-leukemic cells cause RT-PCR false positives, whereas NGS targeting IG/TR rearrangements specifically detects leukemia-derived clones (96, 97).Collectively, these findings underscore that MRD assessment in Ph+ ALL cannot rely solely on BCR::ABL1 quantification, as transcripts may originate from non-leukemic myeloid cells in multilineage disease—a key factor in prognostic discrepancies (98).

## 7 Conclusion

The treatment landscape for Ph+ ALL has undergone a substantial transformation, shifting from a historical reliance on intensive chemotherapy combined with allo-HSCT toward a multidimensional therapeutic strategy driven by TKIs, immunotherapy, and precision medicine. Current clinical efforts are increasingly focused on exploring synergistic, low-intensity regimens that combine chemotherapy with TKIs, bispecific T-cell engagers, or CAR-T therapy to maximize efficacy while reducing treatment-related toxicity.

Accumulating evidence supports individualized decision-making regarding the role of allo-HSCT. Transplantation remains recommended for patients who fail to achieve CMR after induction therapy, those with high tumor burden, TKI-resistant mutations or persistent MRD positivity. Conversely, patients who attain early and sustained CMR may be appropriate candidates for transplant-sparing strategies utilizing TKIs combined with immunotherapy. In the post-transplant setting, MRD-guided TKI maintenance therapy for at least two years is generally recommended, with potential for treatment discontinuation in cases of durable, sustained CMR. Emerging therapeutic avenues include the development of allosteric *BCR::ABL1* inhibitors, agents targeting apoptotic pathways, and rationally designed combination therapies. Future progress will depend on the continued integration of dynamic MRD assessment, comprehensive genomic risk profiling, and novel therapeutic agents within well-designed prospective clinical trials, ultimately enabling further refinement of precision medicine and improved long-term outcomes for patients with Ph+ ALL.
